# Multimorbidity and fluid biomarkers of Alzheimer's disease: a systematic review

**DOI:** 10.1007/s41999-025-01222-y

**Published:** 2025-05-20

**Authors:** Martina Valletta, Marco Canevelli, Francesca Gasparini, Simona Buscarnera, Martina Salzillo, Federico Triolo, Amaia Calderón-Larrañaga, Alessandra Marengoni, Davide Liborio Vetrano, Giulia Grande

**Affiliations:** 1 Aging Research Center, Department of Neurobiology, Care Sciences and Society, Karolinska Institutet and Stockholm University, Stockholm, Sweden; 2 Department of Human Neuroscience, Sapienza University, Rome, Italy; 3 National Centre for Disease Prevention and Health Promotion, Italian National Institute of Health, Rome, Italy; 4 Department of Medicine, Geriatrics Section, University of Padova, Padua, Italy; 5 Stockholm Gerontology Research Center, Stockholm, Sweden; 6 Department of Clinical and Experimental Sciences, University of Brescia, Brescia, Italy

**Keywords:** Multimorbidity, Chronic diseases, Blood, Cerebrospinal fluid, Biomarkers, Alzheimer’s disease

## Abstract

**Purpose:**

This systematic review aimed to summarize the evidence on the association between multimorbidity and fluid biomarkers of Alzheimer’s disease (AD).

**Methods:**

We systematically searched PubMed, Web of Science, and Embase for studies investigating the association between multimorbidity—defined as the co-occurrence of multiple chronic conditions in the same individual—and levels of cerebrospinal fluid (CSF) or blood biomarkers of AD, focusing on the most established AD biomarkers (amyloid-beta, phosphorylated-tau, total-tau, neurofilament light chain, and glial fibrillary acidic protein). Studies were selected following PRISMA guidelines.

**Results:**

Out of 3,104 records, we identified 10 cross-sectional studies. Four studies assessed CSF biomarkers in dementia-free participants with mean age between 61.8 and 66.6 years, yielding mixed findings with no consistent association between multimorbidity and CSF biomarkers. Six studies focused on blood biomarkers in participants with mean age ranging from 66.5 to 76.4 years, five of which included individuals with dementia. Most of these studies reported an association between multimorbidity and elevated blood biomarker levels.

**Conclusions:**

This review suggests a significant association between multimorbidity and AD blood biomarkers in older populations, while the results on CSF are mixed and inconsistent. Further research is needed, particularly longitudinal studies assessing both CSF and blood biomarkers within the same populations.

**Supplementary Information:**

The online version contains supplementary material available at 10.1007/s41999-025-01222-y.

## Key summary points

**Aim:**

This review aimed to summarize the evidence on the association between multimorbidity and levels of CSF and blood biomarkers of Alzheimer´s disease.

**Findings:**

Most studies on blood biomarkers of Alzheimer’s disease reported an association between multimorbidity and elevated biomarker levels. The studies on CSF biomarkers found mixed results.

**Message:**

Given the heterogeneity of studies and participant characteristics, future research should focus on including older populations with higher multimorbidity burden and examining both CSF and blood biomarkers simultaneously to better understand their interrelations and implications for AD diagnosis and treatment.

**Supplementary Information:**

The online version contains supplementary material available at 10.1007/s41999-025-01222-y.

## Introduction

In the last decades, fluid biomarkers have gained increasing importance in dementia research, particularly in Alzheimer’s disease (AD) [1]. Measured in cerebrospinal fluid (CSF) and blood, these biomarkers offer a window into the brain by reflecting the underlying neuropathological changes [2–4]. Among the most studied biomarkers, amyloid-beta 42 (Aβ42) and phosphorylated tau (p-tau) have emerged as core indicators of AD [5] as they accurately reflect the neuropathological hallmarks of the disease [6–8]. Other biomarkers, while not specific to AD, indicate other neuropathological processes that contribute to dementia onset and clinical manifestation, namely neurodegeneration (e.g., total tau, t-tau; and neurofilament light chain, NfL) [9–11] and glial activation (e.g., glial fibrillary acidic protein, GFAP) [12]. 

CSF biomarkers were the first to be implemented in both research and clinical settings and are now a key component of the diagnostic workflow of dementia, especially in specialized centers. However, their widespread use can be limited by the need for a lumbar puncture, an invasive and resource-intensive procedure that remains unavailable in primary care settings. Blood biomarkers offer a less-invasive and more affordable alternative with the potential for a wider application, but more research is needed before their clinical implementation [13].

When interpreting biomarkers’ results, both in CSF and blood, it is essential to consider the factors that can affect their concentrations and potentially require adjustments of the cutoff values to reduce the risk of misclassification. While also true for CSF biomarkers, this may be especially relevant for blood biomarkers, whose production and metabolism can also occur outside the central nervous system [13]. Moreover, the concentration of blood biomarkers may be affected by several non-neurological factors, such as age, sex, ethnicity, and chronic diseases [14–17].

In older populations, where these biomarkers are most likely to be applied, multimorbidity, defined as the coexistence of multiple chronic diseases within the same individual [18], is the norm rather than the exception. Multimorbidity has already been shown to contribute to brain changes [19] and increase the risk of dementia in older adults [20]. This raises important questions about how the presence of multimorbidity might affect biomarker levels and their interpretation. In this systematic review, we aimed to summarize the evidence on the association between multimorbidity and fluid biomarkers of AD.

## Methods

We systematically reviewed studies assessing the association between multimorbidity and levels of fluid (i.e., measured in CSF or blood) biomarkers of AD in adults aged 18 years or older. The protocol of the present study was registered in the international prospective register of systematic reviews PROSPERO (registration number CRD42024594348). This systematic review was reported in accordance with the Preferred Reporting Items for Systematic Reviews and Meta-Analyses (PRISMA) recommendations [21]. For the present study, no ethics committee approval was necessary.

### Data sources and searching

We searched the PubMed electronic database of the National Library of Medicine, the Web of Science database, and Embase for relevant articles published from inception up to the 1st of June 2024. MeSH terms and free words referring to *multimorbidity*, *CSF*, *blood biomarkers*, and *AD* were used as keywords. The detailed search queries are reported as supplementary materials. References from selected papers and other relevant articles were screened for potential additional studies according to a snowball principle.

### Study selection

Four team members (SB, FG, MS, MV) independently screened the titles and abstracts of the retrieved studies. Original studies reporting information on multimorbidity (i.e., defined as the co-occurrence of multiple chronic diseases in the same individual) and its association with the levels of CSF or blood biomarkers of AD were included. In the present study, we focused on the most established AD fluid biomarkers: Aβ, p-tau, t-tau, NfL and GFAP [5, 22]. Articles were excluded if they (a) did not investigate the association between multimorbidity and CSF or blood biomarkers of AD; (b) did not provide a measure of multimorbidity; (c) did not include any of the five abovementioned biomarkers; (d) did not present original data (i.e., reviews, guidelines/recommendations, letters to the editor, commentaries, editorials, case reports); (e) were conducted in vitro or on animals; (f) were written in languages other than English. The full text of the articles selected after titles and abstracts screening was further independently evaluated by the same team members. Any disagreement was resolved through consensus.

### Data extraction

Two team members (MV and MC) independently extracted the information from the selected studies using a standardized form. Any discrepancy was resolved through consensus or involving a third author if necessary. Extracted information included: (a) article information (i.e., first author, year, country); (b) study design, setting, and cohort name; (c) characteristics of the study population (i.e., number of participants, age, sex, cognitive status, ethnicity); (d) multimorbidity definition and operationalization; (e) measured fluid biomarkers; (f) main results; (g) other relevant information (i.e., biomarker transformation, adjustments, correction for multiple comparisons). When specific data required for extraction were unavailable in the published reports, the corresponding author was contacted to obtain the information. When more than one measure of association was provided, the fully adjusted estimate was used for the present study.

### Assessment of risk of bias

Study quality was evaluated independently by two team members (MV and MC) through the Agency for Healthcare Research and Quality (ARHQ) methodology checklist, which is applicable to cross-sectional studies [23, 24]. This tool includes 11 items. Each item receives a score of 1 if the answer is “yes” or 0 if the answer is “no” or “unclear”. The final score thus ranges from 0 (i.e., lowest quality) to 11 (i.e., highest quality). The adopted quality assessment tool is reported as a supplementary material.

### Synthesis of results

Studies that reported presence or absence of association between multimorbidity and CSF/blood biomarkers were represented in a harvest plot. For each bar of the plot, the height represents the number of study participants, and the color represents the investigated biomarker. The results on CSF and blood biomarkers are shown separately. GraphPad Prism 9 was used to create the figure.

## Results

**Fig. 1:**
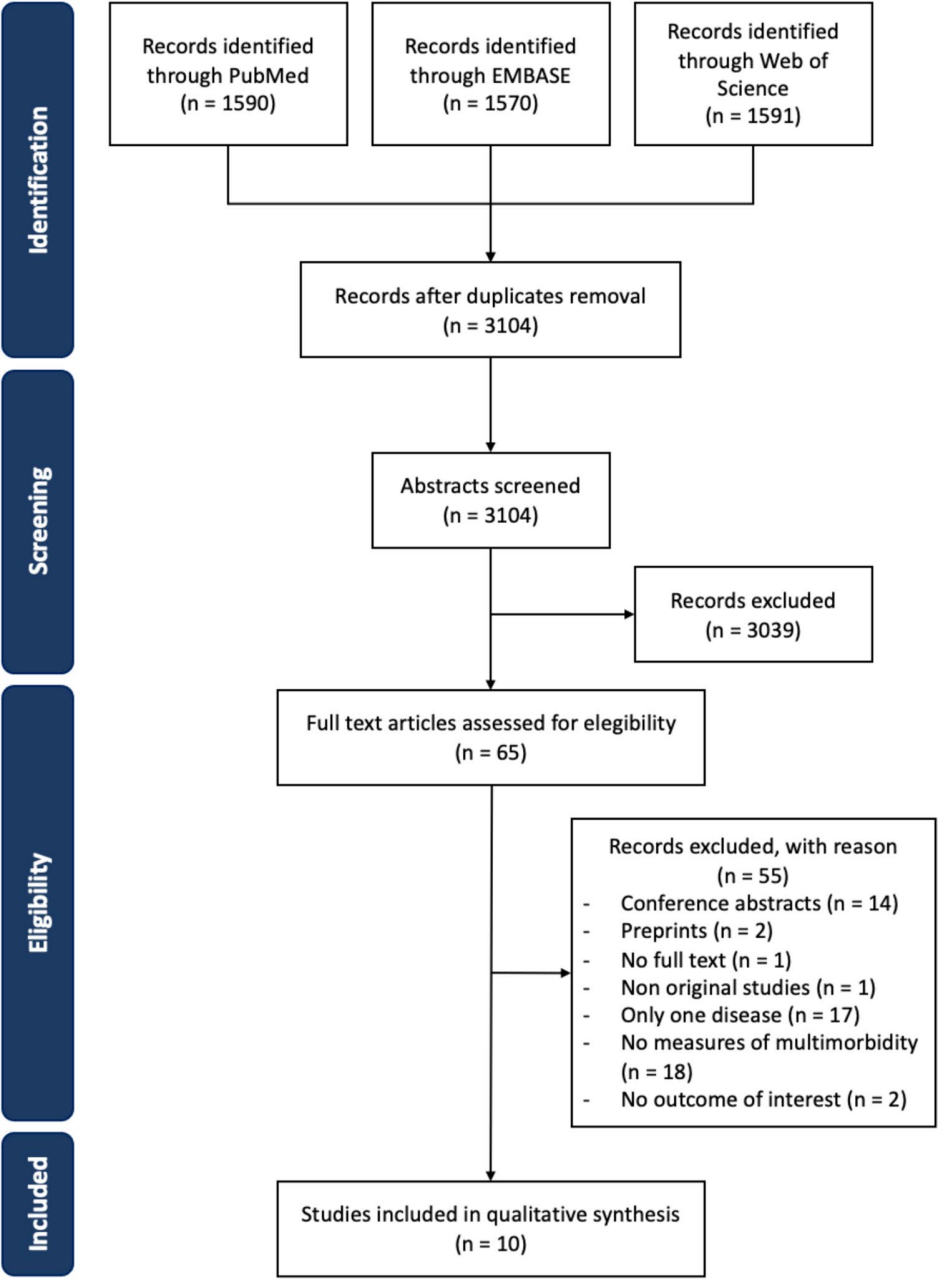
Systematic review flow-chart

Figure 1 shows the PRISMA flow chart of the systematic review. After duplicate removal, a total of 3104 articles were retrieved; 3039 articles were excluded after title and abstract screening, and 55 were excluded after full-text reading (reasons for exclusions are reported in Fig. 1). Ten articles were thus included in the final qualitative assessment [25–34].

### Study description

The main characteristics and the results of the selected studies are summarized in Table 1. The studies were published between 2019 and 2024. Four studies were conducted in Europe, three in the USA, and three in China. All the included studies had a cross-sectional design.

**Table 1:** Characteristics and main findings of the identified studies

A) Studies on CSF biomarkers							
First author, Year, Country	Design of the study, setting and cohort name	Characteristics of the Study Population	Multimorbidity definition, operationalization and prevalence	Biomarkers and type of assay	Results	Note	ARHQ
Li et al. 2024 China [25]	Cross-sectional Participants enrolled from Qingdao Municipal Hospital Chinese Alzheimer’s Biomarker and LifestylE (CABLE)	Han Chinese participants aged 40–90 years Cognitively intact *N* = 1464 Mean age (SD) = 61.80 (10.16) Females, *n* (%) = 629 (42.96)	Participants were categorized based on the number of cardiometabolic diseases (CMD, i.e., hypertension, diabetes, heart diseases and stroke): • CMD-free • single CMD • CMD multimorbidity (≥ 2 CMDs) CMD multimorbidity, *n* (%) = 310 (21.17%)	CSF Aβ40 CSF Aβ42 CSF Aβ42/40 CSF p-tau181 CSF p-tau181/Aβ42 CSF t-tau CSF t-tau/Aβ42 ELISA (Fujirebio)	A greater number of CMD was associated with: • Higher CSF p-tau181 (*β* = 0.078, *p* = 0.044) • Higher CSF t-tau (*β* = 0.030, *p* = 0.047) CMD multimorbidity vs. CMD-free: • Higher CSF p-tau181 (*β* = 0.165, *p* = 0.037) • Higher CSF t-tau (*β* = 0.065, *p* = 0.033) Stratified analyses: the associations between CMD multimorbidity and CSF T-tau levels were found in male and *APOE ε4* non-carriers	The Box-Cox transformation and the *z* score standardization for CSF biomarkers were performed Adjustment for age, sex, education, MMSE, *APOE ε4* carrier status, BMI, cigarette use, alcohol use, and physical activity	9
Stirland et al. 2019 UK [19]	Cross-sectional Participants recruited from multiple existing cohorts (both research and clinical) across Europe European Prevention of Alzheimer’s Dementia (EPAD)	Participants aged over 50 years Dementia-free *N* = 447 Mean age (SD) = 66.6 (6.58) Females, *n* (%) = 234 (52.3)	Count of the total number of chronic conditions for each participant (among 39 considered) Multimorbidity defined as the presence of ≥ 2 chronic conditions Mean number of chronic conditions (SD) = 1.1 (1.27) Multimorbidity, *n* (%) = 128 (28.6)	CSF Aβ42 Electrochemiluminescence (Elecsys)	Each additional comorbid condition was associated with: • Lower likelihood of CSF amyloid positivity (OR = 0.82, 95% CI 0.68–0.97; *p* = 0.026) • Higher CSF Aβ42 (*β* = 52.4, 95% CI 9.9–98.5, *p* = 0.017) Multimorbidity vs no multimorbidity: • Lower likelihood of CSF amyloid positivity (OR = 0.59, 95% CI 0.37–0.95, *p* = 0.030)	Adjustment for age, sex, education, *APOE ε4* carrier status, family history of dementia	7
Aerqin et al. 2024 China [24]	Cross-sectional Participants enrolled from Qingdao Municipal Hospital Chinese Alzheimer’s Biomarker and LifestylE (CABLE)	Han Chinese participants aged 40–90 years Dementia-free *N* = 1402 (1276 CU and 126 MCI) Mean age (SD) = 62.33 ± 10.34 Females, *n* (%) = 569 (40.6)	Categories of multimorbidity: • Non multimorbidity: 0 or 1 conditions • Multimorbidity: 2 or 3 conditions • Severe multimorbidity: 4 or more conditions Multimorbidity patterns identified using fuzzy c-means cluster analysis: • Unspecific • Metabolic • Degenerative Multimorbidity, *n* (%) = 975 (69.5)	CSF Aβ40 CSF Aβ42 CSF Aβ42/40 CSF p-tau CSF p-tau/Aβ42 CSF t-tau CSF t-tau/Aβ42 ELISA (Fujirebio)	Severe multimorbidity vs non-multimorbidity: • Lower CSF Aβ42 (*β* = − 0.226, pFDR = 0.012) • Lower CSF Aβ42/40 (*β* = − 0.248, pFDR = 0.009) • Higher CSF p-tau/Aβ42 (*β* = 0.239, pFDR = 0.018) • Higher t-tau/Aβ42 (*β* = 0.211, pFDR = 0.048) APOE-stratified analyses: the associations were more evident in ε4 non-carriers Metabolic vs. unspecific pattern: • Higher CSF Aβ40 (*β* = 0.159, pFDR = 0.036) • Higher CSF p-tau (*β* = 0.132, pFDR = 0.035) • Higher CSF t-tau (*β* = 0.126, pFDR = 0.035) *APOE*-stratified analyses: in ε4 non-carriers, the metabolic pattern was associated with higher CSF Aβ40 Analyses stratified by cognitive status (CU and MCI): no findings in MCI group	CSF biomarkers were log-transformed Adjustment for age, sex, education, and *APOE ε4* carrier status Benjamini Hochberg false discovery rates (FDR) correction	8
Zenuni et al. 2021 Italy [28]	Cross-sectional Participants enrolled from the Neurology Unit of Tor Vergata University Hospital (Rome, Italy)	Cognitively intact *N* = 55 Mean age (SD) = 66.4 (13.0) Females, % = 56	Charlson Comorbidity Index (CCI) Mean CCI (SD) = 4.0 (2.1)	CSF Aβ40 CSF Aβ42 CSF p-tau181 CSF Aβ42/p-tau181 CSF t-tau Chemiluminescent immunoassay (CLIA) (Lumipulse, Fujirebio) for Aβ42 and t-tau; ELISA for Aβ40 and p-tau	No significant associations between CCI and biomarkers	Linear model adjusted for age and sex	4

B) Studies on blood biomarkers							
First author, Year, Country	Design of the study, setting and cohort name	Characteristics of the study population	Multimorbidity definition and operationalization	Biomarkers	Results	Note	ARHQ
Syrjanen et al. 2022 USA [29]	Cross-sectional Population-based study Mayo Clinic Study of Aging (MCSA)	Participants aged 50 years and over, 99% white CU and MCI/dementia *N* = 996 (859 CU, 133 MCI, and four dementia) Median age (Q1, Q3) = 76.39 (68.26, 81.88) Females, *n* (%) = 437 (43.9)	CCI Median CCI (Q1, Q3) = 2 (1,4)	Plasma Aβ40 Plasma Aβ42 Plasma Aβ42/40 Plasma t-tau Plasma NfL Simoa (Quanterix)	Among CU participants CCI was associated with: • Higher plasma Aβ40 (*β* = 0.06, 95% CI 0.04–0.08) • Higher Aβ42 (*β* = 0.06, 95% CI 0.04–0.09) • Higher Aβ42/40 ratio (*β* = 0.03, 95% CI 0.01–0.05) • Higher t-tau (β = 0.04, 95% CI 0.02–0.07) • Higher NfL (*β* = 0.05, 95% CI 0.03–0.07) Among MCI/dementia participants CCI was associated with: • Higher Aβ40 (*β* = 0.06, 95% CI 0.01–0.11)	Plasma biomarkers were *z*-scored Adjustment for age and sex	8
Valletta et al. 2024 Sweden [30]	Cross-sectional Population-based study Swedish National study on Aging and Care in Kungsholmen (SNAC-K)	Participants aged 60 years and over Dementia-free *N* = 2366 Median age (Q1, Q3) = 72.2 (60.9, 81.1) Females, *n* (%) = 1461 (61.7)	Categories of multimorbidity: • 0 or 1 disease • 2 or 3 diseases • 4 or 5 diseases • 6 or more diseases Median number of chronic diseases (Q1, Q3) = 3.0 (2.0–5.0)	Serum Aβ42/40 Serum p-tau181 Serum T-Tau Serum NfL Serum GFAP Simoa (Quanterix)	4–5 chronic diseases vs 0 or 1: • Higher serum p-tau181 (*β* = 0.19, 95% CI 0.12—0.27) • Higher serum t-tau (*β* = 0.13, 95% CI 0.02—0.23) • Higher serum NfL (*β* = 0.13, 95% CI 0.07—0.19) • Higher serum GFAP (*β* = 0.03, 95% CI 0.00—0.07) 6 chronic diseases vs 0 or 1: • Higher serum p-tau181 (*β* = 0.23, 95% CI 0.13—0.33) • Higher serum t-tau (*β* = 0.20, 95% CI 0.06—0.34) • Higher serum NfL (*β* = 0.32, 95% CI 0.25—0.40) • Higher serum GFAP (*β* = 0.09, 95% CI 0.04—0.13) The associations did not change after the exclusion of participants MMSE < 27	Plasma biomarkers were z-scored Adjustment for age, sex and education	8
O’Bryant et al. 2022 USA [31]	Cross-sectional Population-based study Health & Aging Brain among Latino Elders (HABLE)	Mexican American (*N* = 890) and non-Hispanic White (*N* = 813) participants CU, MCI and dementia *N* = 1705 (1351 CU, 243 MCI and 111 dementia) Mean age (SD) = 66.49 (8.76) Females, % = 60	Comorbidity index created by combining presence/absence of three conditions (i.e., hypertension, diabetes, dyslipidemia): • None • Only one • Two • All three Comorbidity index ≥ 2, *n* (%) = 795 (49)	Plasma NfL Simoa (Quanterix)	A higher comorbidity index was associated with: • Higher plasma NfL (*β* = 1.45, *p* < 0.001) Ethnicity-stratified analyses: the comorbidity index was not significant among non-Hispanic whites (*β* = 0.69, *p* = 0.094), whereas it was highly significant among Mexican Americans (β = 2.35, *p* < 0.001)	Adjustment for age and sex	7
Sarto et al. 2024 Spain [32]	Cross-sectional Participants enrolled from the Alzheimer’s disease and other cognitive disorders unit, Hospital Clínic de Barcelona (Barcelona, Spain)	Participants with suspected cognitive impairment and CU volunteers 360 participants (36 CU, 67 SND, 174 AD, 21 LBD and 62 FTD) Mean age (SD) = 66.5 (7.7) Females, % = 55	CCI Multimorbidity, % = 47%	Plasma p-tau181 Plasma NfL Plasma GFAP Simoa (Quanterix)	Higher CCI was associated with: • Higher plasma NfL • Higher plasma GFAP After further adjustment for CSF or PET Aβ status only the association between CCI and GFAP remained significant (β = 0.179, 95%CI 0.065 − 0.285, *p* = 0.002)	Plasma biomarkers were log-transformed and *z*-scored Age and sex adjustment The associations that were significant were further adjusted for CSF or PET Aβ status	7
Ren et al. 2024 China [33]	Cross-sectional Population-based study Multimodal Interventerventions to Delay Dementia and Disability in Rural China (MIND-China)	Rural-dwelling Chinese older adults aged 60 years and over CU, MCI and AD Total sample N = 5432 Subsample with plasma biomarkers N = 1412 (887 CU, 391 MCI, 134 AD) Mean age (SD) of the total sample = 70.71 (5.73) Females, *n* (%) in the total sample = 3121 (57.5)	Multimorbidity defined as the presence of ≥ 2 among 23 chronic health conditions Hierarchical clustering method to generate clusters of multimorbidity: • Metabolic • Cardiac-musculoskeletal (MSK) • Degenerative- ocular • Respiratory • Mixed Multimorbidity, n (%) = 830 (58.8)	Plasma Aβ40 Plasma Aβ42 Plasma t-tau Plasma NfL Simoa (Quanterix)	An increasing number of chronic diseases was associated with • higher plasma Aβ40 (*β* = 0.054, 95% CI 0.013–0.096) • higher plasma Aβ42 (*β* = 0.058, 95% CI 0.017–0.100) • higher NfL (*β* = 0.062, 95% CI 0.023–0.101) Multimorbidity vs no multimorbidity: • higher plasma Aβ42 (*β* = 0.108, 95% CI 0.001–0.215) • higher plasma NfL (*β* = 0.108, 95% CI 0.008–0.208) not significant after Bonferroni correction Metabolic cluster: • higher plasma NfL (*β* = 0.120, 95% CI 0.018–0.221), not significant after Bonferroni correction Degenerative ocular cluster: • higher plasma NfL (*β* = 0.333, 95% CI 0.102–0.564) Cardiac-MSK cluster: • higher plasma Aβ42 (*β* = 0.147, 95% CI 0.024–0.270), not significant after Bonferroni correction	Plasma biomarkers that were not normally distributed were log-transformed. All the plasma biomarkers were *z*-scored Adjustment for age, sex, education, current smoking, alcohol consumption, and physical inactivity Bonferroni correction performed to adjust for multiple comparisons	9
Mielke et al. 2022 USA [34]	Cross-sectional Population-based study Mayo Clinic Study of Aging (MCSA)	Participants aged 30 to 98 years, 96.8% white CU and MCI/dementia N = 1329 (1161 CU, 153 MCI, and 15 dementia) Median age (Q1, Q3): 73.2 (53.5, 81.3) Females, n (%) = 599 (45.1)	CCI Median CCI (Q1, Q3) = CCI 2 (0, 4)	Plasma p-tau181 Plasma p-tau217 Electrochemiluminescence, Meso Scale Discovery platform (Lilly)	Among CU participants CCI was associated with: • Higher plasma p-tau181 (*β* per 5 = 0.16, 95% CI 0.10–0.21) • Higher plasma p-tau217 (*β* per 5 = 0.12, 95% CI 0.06–0.17) No association was found in the MCI/dementia group	Plasma biomarkers were z-scored Adjustment for age and sex	9

*Aβ*40 amyloid beta-40, *Aβ*42 amyloid beta-42, *AD* = Alzheimer’s disease, *APOE* apolipoprotein E, *ARHQ* Agency for Healthcare Research and Quality (ARHQ) methodology checklist, *BMI* body mass index, *CCI* Charlson Comorbidity Index, *CMD* cardiometabolic diseases, *CSF* cerebrospinal fluid, CU cognitively unimpaired, *ELISA* enzyme-linked immunosorbent assay, *FDR* false discovery rate, *GFAP* glial fibrillary acidic protein, *MCI* mild cognitive impairment, *MMSE* mini mental state examination, *MSK* musculoskeletal, *NfL* neurofilament light chain, *PET* positron emission tomography, *P*-*tau*181 phosphorylated tau 181, *P*-*tau*217 phosphorylated tau 217, Simoa single-molecule array, *T-Tau* total tau

Four of the ten selected studies assessed AD biomarkers in CSF [25–28]. The population of these studies ranged from 55 [28] to 1464 [25] participants. The mean age of study participants was between 61.8 and 66.6 years and the proportion of females was between 40.6 and 56.0%. All four studies excluded participants with dementia.

The other six studies investigated AD blood biomarkers [29–34]. They included between 360 [32] and 2366 participants [30], with mean/median age ranging from 66.5 to 76.4 years and a proportion of females from 43.9% to 61.7%. Five studies included participants with dementia [29, 31–34]. Five out of the six studies were population-based [29–31, 33, 34].

### Fluid biomarkers measurement

Three of the four studies on CSF AD biomarkers assessed Aβ40, Aβ42, p-tau, and t-tau [25, 27, 28], while one only considered Aβ42 [26]. All the studies on CSF biomarkers used immunoassays for biomarker determination, either enzyme-linked immunosorbent assay (ELISA) [25, 27, 28], electrochemiluminescence [26] or chemiluminescent immunoassay (CLIA) [28]. In all the studies on blood biomarkers, the biomarkers were measured on plasma, except for one study in which they were measured on serum [30]. Four of the studies on blood biomarkers considered multiple biomarkers, while two focused on one single biomarker, which in one case was NfL [31] and in the other was p-tau [34]. All the studies exploring blood biomarkers used single-molecule array (Simoa) for biomarker quantification [29–33], except for one that used electrochemiluminescence on a Meso Scale Discovery platform [34]. No study explored both CSF and blood biomarkers.

### Multimorbidity assessment

For multimorbidity assessment, two of the studies on CSF biomarkers considered the count of chronic diseases [26, 27], one used the Charlson Comorbidity Index (CCI) [28] and one focused exclusively on cardiometabolic diseases (i.e., hypertension, diabetes, heart disease, and stroke) [25]. Among the studies on blood biomarkers, three used the CCI [29, 32, 34], two considered the count of chronic diseases [30, 33] and one considered only three chronic conditions (i.e., hypertension, diabetes, dyslipidemia) [31]. One study on CSF [27] and one study on blood [33] biomarkers also investigated data driven multimorbidity patterns.

### Risk of bias

The average ARHQ score of the 10 included studies was 7.6. The risk of bias was lower for studies on blood than for studies on CSF biomarkers (mean ARHQ score 8 vs 7). All the studies had an ARHQ score between 7 and 9, except for one study on CSF biomarkers whose ARHQ was lower, indicating a higher risk of bias [28].

### Multimorbidity and CSF biomarkers of AD

**Fig. 2:**
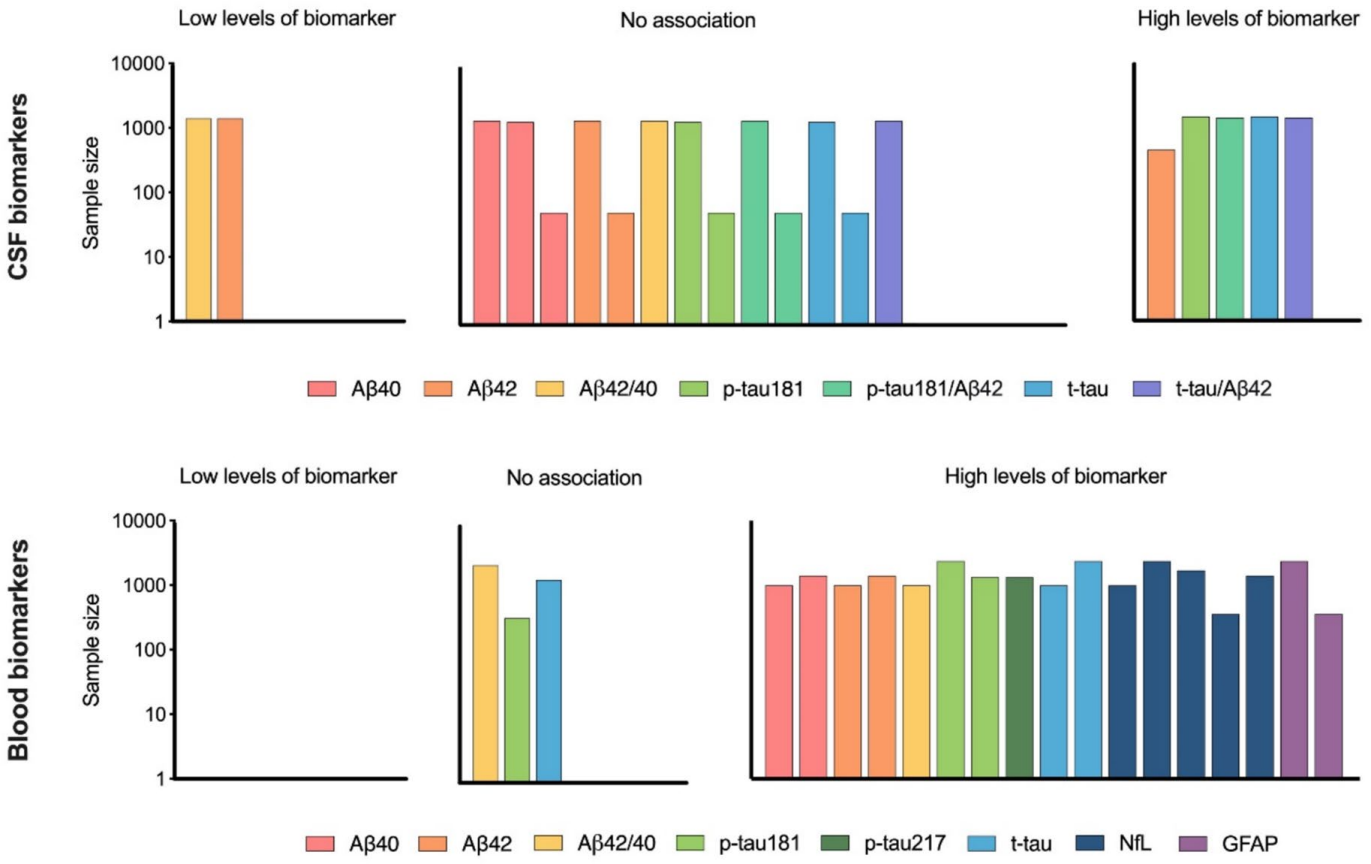
Association between multimorbidity and fluid biomarkers of Alzheimer’s disease. Bar Heights: The height of each bar represents the study’s sample size (log-transformed). Different colors are used to represent each biomarker. Biomarker Categories: Results are displayed separately for cerebrospinal fluid (CSF) biomarkers and blood biomarkers

Figure 2 depicts the studies reporting the presence or absence of an association between multimorbidity and CSF biomarkers of AD. None of the three studies found an association between multimorbidity and CSF Aβ40 [25, 27, 28]. The studies investigating the association between multimorbidity and the other CSF biomarkers yielded mixed results although the majority of findings tended to be null (Fig. 2). Considering multimorbidity patterns, one study conducted on participants of the CABLE study found higher levels of CSF Aβ40, CSF p-tau, and CSF t-tau in individuals with the metabolic pattern compared to those with unspecific multimorbidity (i.e., a group in which no disease was over-expressed compared to the overall population prevalence) [27]. Similarly, the other study conducted on the CABLE population found an association between cardiometabolic multimorbidity and elevated levels of CSF p-tau181 and CSF t-tau [25].

### Multimorbidity and blood biomarkers of AD

Most studies on blood biomarkers of AD consistently reported an association between multimorbidity and elevated levels of the biomarkers (Fig. 2). The only discrepancies were found for the Aβ ratio, for which one study reported an association [29] and one did not [30], and for p-tau181 and t-tau, where two studies reported an association [29, 30, 34] and another did not [32, 33]. One study also explored the association between multimorbidity patterns and plasma biomarkers of AD and found elevated levels of NfL in the metabolic and in the degenerative-ocular (i.e., which included cataract and glaucoma) patterns [33]. Elevated levels of Aβ42 were found in the cardiac–musculoskeletal pattern, which was characterized by heart diseases, degenerative disc disease and arthritis [33].

## Discussion

Based on a systematic search of the literature, we identified ten studies that examined the association between multimorbidity and fluid biomarkers of AD. Among these, four studies focused on CSF biomarkers, while six investigated blood biomarkers. The included studies varied widely in terms of setting (e.g., clinical versus population-based studies), characteristics of study participants (e.g., inclusion or exclusion of individuals with dementia), and operationalization of multimorbidity. Additionally, the assessed biomarkers across studies were inconsistent. Due to the scarcity and the heterogeneity of the available evidence, a quantitative synthesis of the results was not possible.

The available evidence indicates an association between the presence of multimorbidity and the elevated levels of multiple biomarkers of AD in the blood. In contrast, the results related to CSF biomarkers were mixed, with no consistent association emerging between multimorbidity and biomarker levels. This discrepancy may indicate that CSF biomarkers, which more closely reflect central neuropathology, are likely to be less influenced by the presence of multimorbidity. However, given that none of the included studies simultaneously explored both CSF and blood biomarkers, direct comparisons are not possible due to differences in study settings and characteristics of the study populations.

Several factors complicate the interpretation of the lack of association between multimorbidity and CSF biomarkers. These include relatively small sample sizes, heterogeneity in measuring multimorbidity, and, most importantly, the characteristics of study participants. A notable limitation of the CSF studies is the relatively young mean age of participants, which ranged between 61.8 and 66.6 years. While this age range represents a critical period for AD research, it may not accurately represent the multimorbidity burden prevalent in older populations. For instance, the prevalence of multimorbidity was below 30% in two [25, 26] of the four studies on CSF biomarkers, while a previous systematic review reported a prevalence of multimorbidity in the older population ranging from 55 to 98% [35]. Although prevalence estimates of multimorbidity vary greatly depending on the definitions used, there is wide consensus around the fact that both the prevalence and severity of multimorbidity increase with advancing age [18, 35–37]. The enrollment of a relatively young study population may thus limit the capability to detect significant associations between multimorbidity and CSF biomarkers of AD. Moreover, most studies on CSF biomarkers were conducted in clinical settings and likely included healthier and more selected study populations than those on blood biomarkers. It is plausible that participants enrolled in the studies on CSF biomarkers had milder multimorbidity, as individuals with more complex clinical profiles, including severe or poorly controlled chronic conditions, may have been excluded from lumbar puncture procedures. Future research should prioritize older populations with higher multimorbidity burden to enhance the potential for uncovering significant interactions between multimorbidity, neuropathology, and CSF biomarkers.

Based on our systematic review, studies investigating blood biomarkers, as compared to those on CSF ones, reported a clearer association between multimorbidity and elevated biomarker levels, particularly for p-tau, t-tau, and NfL. The concentration of blood biomarkers may be more affected by chronic diseases due to their production, distribution, or elimination outside the central nervous system [14, 17]. For instance, previous studies hypothesized that the association between chronic kidney disease and elevated levels of AD blood biomarkers might be due to reduced renal clearance [17] while the variations of blood biomarkers levels with body mass index might be due to variations in their blood distribution [14]. It is also worth noting that many studies have shown that individuals with multimorbidity, compared to their non-multimorbid peers, experience accelerated brain aging [19, 38] and have a higher risk of dementia [39–42]. Several individual diseases have been linked to neuropathological changes. For example, heart diseases have been associated with cerebrovascular pathology and neurodegeneration [43], and anemia has been linked to cerebral hypoxia and brain atrophy [44, 45]. The increased levels of biomarkers in the bloodstream could therefore also be a reflection, at least in part, of the neuropathology related to multimorbidity.

Beyond the count of diseases, few studies have explored how the levels of some AD fluid biomarkers vary in the presence of specific combinations of diseases [25, 27, 31, 33]. In particular, variations in the levels of both CSF and blood biomarkers have been consistently reported in individuals with metabolic or cardiovascular multimorbidity [25, 27, 31, 33]. These findings are not surprising, since, as mentioned above, metabolic and cardiovascular diseases are well-known risk factors for accelerated brain aging and dementia [43], and could potentially reflect a greater brain pathology associated with these conditions. Evidence on other disease combinations or multimorbidity patterns is instead more limited.

The shift towards a biological definition of AD [5] requires the adoption of biomarkers with proven clinical validity and utility [46]. Studying the impact of key aging-related syndromes, such as multimorbidity, on the concentration and accuracy of fluid biomarkers of AD is a critical step in the roadmap for their validation [46]. This carries important clinical implications as the variations in the levels of fluid biomarkers of AD due to chronic diseases may confound AD diagnosis and hinder the identification of patients who might benefit from disease-modifying treatments. Specifically, these variations may increase the risk of false-positive diagnoses, where individuals with a high burden of chronic diseases may be misdiagnosed with AD. Notably, we found only one study exploring the impact of multimorbidity on plasma p-tau217, which has recently been proposed as a standalone biomarker for the diagnosis of AD [5]. Furthermore, studying the association between multimorbidity and CSF and blood biomarkers has relevant research implications offering insights into the biological basis of the relationship between multimorbidity and dementia and potentially fostering the identification of new therapeutic targets.

### Strengths and limitations

The strengths of this review include the adoption of a comprehensive and reproducible search strategy and the use of three different electronic databases, which ensured broad coverage of relevant studies. Moreover, the study selection and data extraction were conducted by independent researchers to minimize the risk of bias. The main limitation of the review is scarcity and heterogeneity of the retrieved studies, which made a quantitative synthesis of the evidence not possible.

### Conclusions and future directions

This review underscores the need for more research to clarify the relationship between multimorbidity and fluid biomarkers of AD, particularly in the premise of their clinical implementation. Blood biomarkers hold significant potential for widespread use due to their accessibility. However, their susceptibility to influence beyond neuropathology, particularly in older adults with complex clinical profiles, warrants further investigations. Future studies should prioritize longitudinal designs to better understand how the long-term trajectories of multimorbidity, biomarkers and cognitive decline are related. Additionally, future studies should explore the relationship between fluid biomarkers of AD and other measures of somatic burden, including frailty. Finally, exploring both CSF and blood biomarkers within the same study population would provide valuable insights into how these biomarkers compare and correlate with the presence of multimorbidity. 

## Supplementary Information

Below is the link to the electronic supplementary material.

Supplementary file1 (DOCX 18 kb)
