# Supplementary material for: Multimorbidity and fluid biomarkers of Alzheimer's disease: a systematic review
Source: Eur Geriatr Med. 2025 May 20;16(4):1121–36. doi: 10.1007/s41999-025-01222-y (PMC12378289; doi:10.1007/s41999-025-01222-y)
Supplement: Supplementary file 1 — Supplementary file1 (DOCX 18 kb) [file 41999_2025_1222_MOESM1_ESM.docx]

**SUPPLEMENTARY MATERIAL**

**Search queries**

**Pubmed**

("multimorbidity"[MeSH] OR "Comorbidity"[Mesh] OR "multimorbid*"[Title/Abstract] OR "multi-morbid*"[ Title/Abstract] OR "comorbid*"[ Title/Abstract] OR "co-morbidit*"[ Title/Abstract] OR "disease pattern*"[ Title/Abstract] OR "disease cluster*"[ Title/Abstract] OR "coexisting diseases*"[ Title/Abstract] OR " chronic disease*"[ Title/Abstract] OR " chronic condition*"[ Title/Abstract])

AND

("Biomarkers/blood"[Mesh] OR "Biomarkers/cerebrospinal fluid"[Mesh] OR "Neurofilament Proteins/blood"[Mesh] OR "Neurofilament Proteins/cerebrospinal fluid"[Mesh] OR "tau Proteins/blood"[Mesh] OR "tau Proteins/cerebrospinal fluid"[Mesh] OR "Amyloid/blood"[Mesh] OR "Amyloid/cerebrospinal fluid"[Mesh] OR "Glial Fibrillary Acidic Protein/blood"[Mesh] OR "Glial Fibrillary Acidic Protein/cerebrospinal fluid"[Mesh] OR "blood biomarker*"[Title/Abstract] OR "blood-based biomarker*"[Title/Abstract] OR "serum biomarker*"[Title/Abstract] OR "plasm* biomarker*"[Title/Abstract] OR "cerebrospinal fluid biomarker*"[Title/Abstract] OR "csf biomarker*"[Title/Abstract] OR "fluid biomarker*"[Title/Abstract] OR "nfl"[Title/Abstract] OR "neurofilament"[Title/Abstract] OR "amyloid*"[Title/Abstract] OR "tau"[Title/Abstract] OR "gfap*"[Title/Abstract] OR "glial fibrillary acidic protein"[Title/Abstract])

AND

("Alzheimer Disease"[Mesh] OR "Dementia"[Mesh] OR "Neurodegenerative Diseases"[Mesh] OR "Neurocognitive Disorders"[Mesh] OR "Alzheimer*"[Title/Abstract] OR "dementia*"[Title/Abstract] OR "neurodegenerat*"[Title/Abstract] OR “neurocognitive disorder*"[Title/Abstract] OR "cognit*"[Title/Abstract])

**Web of science**

TS=(("multimorbid*" OR "multi-morbid*" OR "comorbid*" OR "co-morbidit*" OR "disease pattern*" OR "disease cluster*" OR "coexisting diseases*" OR " chronic disease*" OR " chronic condition*") AND ("blood biomarker*" OR "blood-based biomarker*" OR "serum biomarker*" OR "plasm* biomarker*" OR "cerebrospinal fluid biomarker*" OR "csf biomarker*" OR "fluid biomarker*" OR "nfl" OR "neurofilament" OR "amyloid*" OR "tau" OR "gfap*" OR "glial fibrillary acidic protein") AND ("Alzheimer*" OR "dementia*" OR "neurodegenerat*" OR “neurocognitive disorder*" OR "cognit*"))

**EMBASE**

| #22 | #18 AND #19 AND #20 AND #21 |
| --- | --- |
| #21 | #15 OR #16 OR #17 |
| #20 | #9 OR #10 OR #11 OR #12 OR #13 OR #14 |
| #19 | #4 OR #5 OR #6 OR #7 OR #8 |
| #18 | #1 OR #2 OR #3 |
| #17 | 'blood':ti,ab,kw OR 'blood-based':ti,ab,kw OR 'serum':ti,ab,kw OR 'plasm*':ti,ab,kw OR 'cerebrospinal fluid':ti,ab,kw OR 'csf':ti,ab,kw OR 'fluid':ti,ab,kw |
| #16 | 'cerebrospinal fluid'/de |
| #15 | 'blood'/de |
| #14 | 'biomarker*':ti,ab,kw OR 'biological marker*':ti,ab,kw OR 'nfl':ti,ab,kw OR 'neurofilament':ti,ab,kw OR 'amyloid*':ti,ab,kw OR 'tau':ti,ab,kw OR 'gfap*':ti,ab,kw OR 'glial fibrillary acidic protein':ti,ab,kw |
| #13 | 'glial fibrillary acidic protein'/de |
| #12 | 'amyloid beta protein'/de |
| #11 | 'tau protein'/de |
| #10 | 'neurofilament'/de |
| #9 | 'biological marker'/de |
| #8 | 'alzheimer*':ti,ab,kw OR 'dementia*':ti,ab,kw OR 'neurodegenerat*':ti,ab,kw OR 'neurocognitive disorder*':ti,ab,kw OR 'cognit*':ti,ab,kw |
| #7 | 'cognitive defect'/de |
| #6 | 'degenerative disease'/de |
| #5 | 'alzheimer disease'/de |
| #4 | 'dementia'/de |
| #3 | 'multimorbid*':ti,ab,kw OR 'multi-morbid*':ti,ab,kw OR 'comorbid*':ti,ab,kw OR 'co-morbidit*':ti,ab,kw OR 'disease pattern*':ti,ab,kw OR 'disease cluster*':ti,ab,kw OR 'coexisting diseases*':ti,ab,kw OR 'chronic disease*':ti,ab,kw OR 'chronic condition*':ti,ab,kw |
| #2 | 'comorbidity'/de |
| #1 | 'multiple chronic conditions'/de |

**Quality assessment tool**

**The** **Agency for Healthcare Research and Quality Methodology Checklist for Cross-Sectional/Prevalence Study**

Website: <http://www.ncbi.nlm.nih.gov/books/NBK35156/>

|  | Question | Yes | No | Unclear |
| --- | --- | --- | --- | --- |
| 1 | Define the source of information (survey, record review) |  |  |  |
| 2 | List inclusion and exclusion criteria for exposed and unexposed subjects (cases and controls) or refer to previous publications |  |  |  |
| 3 | Indicate time period used for identifying patients |  |  |  |
| 4 | Indicate whether or not subjects were consecutive if not population-based |  |  |  |
| 5 | Indicate if evaluators of subjective components of study were masked to other aspects of the status of the participants |  |  |  |
| 6 | Describe any assessments undertaken for quality assurance purposes (e.g., test/retest of primary outcome measurements) |  |  |  |
| 7 | Explain any patient exclusions from analysis |  |  |  |
| 8 | Describe how confounding was assessed and/or controlled |  |  |  |
| 9 | If applicable, explain how missing data were handled in the analysis |  |  |  |
| 10 | Summarize patient response rates and completeness of data collection |  |  |  |
| 11 | Clarify what follow-up, if any, was expected and the percentage of patients for which incomplete data or follow-up was obtained |  |  |  |
